# High nutrient intake during the early postnatal period accelerates skeletal muscle fiber growth and maturity in intrauterine growth-restricted pigs

**DOI:** 10.1186/s12263-018-0612-8

**Published:** 2018-07-27

**Authors:** Liang Hu, Fei Han, Lin Chen, Xie Peng, Daiwen Chen, De Wu, Lianqiang Che, Keying Zhang

**Affiliations:** Institute of Animal Nutrition, Key Laboratory of Animal Disease-Resistance Nutrition, Sichuan Agricultural University, Ministry of Education, No.211 Huimin Road, Wenjiang District, Chengdu, 611130 Sichuan People’s Republic of China

**Keywords:** Birth weight, Nutrient intake, Skeletal muscle, Metabolic status, Pigs

## Abstract

**Background:**

Intrauterine growth-restricted (IUGR) neonates impair postnatal skeletal muscle growth. The aim of this study was to investigate whether high nutrient intake (HNI) during the suckling period could improve muscle growth and metabolic status of IUGR pigs.

**Methods:**

Twelve pairs of IUGR and normal birth weight (NBW) pigs (7 days old) were randomly assigned to adequate nutrient intake and HNI formula milk groups. Psoas major (PM) muscle sample was obtained after 21 days of rearing.

**Results:**

IUGR decreased cross-sectional areas (CSA) and myofiber numbers, activity of lactate dehydrogenase (LDH), and mRNA expression of insulin-like growth factor 1 (IGF-1), IGF-1 receptor (IGF-1R), mammalian target of rapamycin (mTOR), ribosomal protein s6 (RPS6), eukaryotic translation initiation factor 4E (eIF4E), protein expression of phosphorylated mTOR (P-mTOR), and phosphorylated protein kinase B (P-Akt) in the PM muscle of pigs. Irrespective of birth weight, HNI increased muscle weight and CSA, the concentration of RNA, and ratio of RNA to DNA, as well as ratio of LDH to β-hydroxy-acyl-CoA-dehydrogenase in the PM muscle of pigs. Furthermore, HNI increased percentages of MyHC IIb, mRNA expression of IGF-1, IGF-1R, Akt, mTOR, RPS6, and eIF4E, as well as protein expression of P-mTOR, P-Akt, P-RPS6, and P-eIF4E in the PM muscle of pigs.

**Conclusion:**

The present findings suggest that high nutrient intake during the suckling period could improve skeletal muscle growth and maturity, which is associated with increasing the expression of protein deposition-related genes and accelerating the development of glycolytic-type myofiber in pigs.

**Electronic supplementary material:**

The online version of this article (10.1186/s12263-018-0612-8) contains supplementary material, which is available to authorized users.

## Background

Epidemiological studies have demonstrated that intrauterine growth-restricted (IUGR) neonates are associated with the higher morbidity and mortality during the early life period, as well as a greater risk of the metabolic syndrome in adult life [1, 2]. Furthermore, previous studies showed that IUGR had negative impacts on the growth and development of skeletal muscle in pigs, including reduced skeletal muscle mass [3] and total myofiber number [4], increased cross-sectional area (CSA) [5], and abnormal lipid deposition [6]. Skeletal muscle plays an important role in the metabolic homeostasis [7], and a defect in normal muscle development during the early postnatal period can permanently alter the subsequent muscle growth, contractile performance, and metabolism [8]. Therefore, improving skeletal muscle development of neonates with IUGR would be beneficial.

Postnatal muscle growth is mostly determined by the total myofiber number and fiber CSAs [9], as well as controlled by many signaling pathways in vivo [10]. Among those, the IGF1-Akt-mammalian target of rapamycin (mTOR) pathway acts as a major positive regulator of muscle growth [11]. Our previous study found high nutrient intake (HNI) could lead to catch-up growth during the suckling period in IUGR pigs [12]; nevertheless, it is not clear whether HNI affects muscle growth and muscle growth-related molecular signal pathway. Since skeletal muscle accounts for about 50% of body mass and approximately 25% of the basal metabolic rate [13], changes in metabolic properties of muscle in the neonatal phase are also correlated with early muscle growth and myofiber maturation [14]. Thus, understanding the effects of HNI during the early postnatal period on the muscular metabolic status may provide new insights into the fiber development of IUGR.

Due to the physiological and genomic similarities between pigs and humans, pigs have been recognized as an excellent experimental model for the study of clinical nutrition [15]. Moreover, pigs exhibit severe naturally occurring IUGR [16]. In the present study, we investigated the effects of HNI on skeletal muscle growth, metabolic status, and the expressions of muscle growth and development-related genes of IUGR pigs.

## Methods

The experiment followed the actual law of animal protection and was approved by the Animal Care and Use committee of Sichuan Agricultural University and performed in accordance with the National Research Council’s Guide for the Care and Use of Laboratory Animals.

### Animals, experimental design, and formula milk

In the current study, we collected the samples from the same animal experiment of our previous study. The experimental pigs and diets were detailed described in our previous study [12]. In brief, 12 pairs of IUGR (~ 0.87 kg) and normal birth weight (NBW, ~ 1.52 kg) pigs (Duroc × (Landrace × Yorkshire)) from 12 healthy sows were selected and all pigs were moved to be individually fed in nursing cages (0.8 m × 0.7 m × 0.4 m) when they were 7 days old (~ 1.68 for IUGR vs. ~ 2.78 kg for NBW). For nutritional treatments, six pairs of NBW and IUGR pigs were allocated to have adequate nutrient intake (ANI), while other six pairs of NBW and IUGR pigs were allocated to have high nutrient intake (HNI). This produced four experimental groups (birth weight/nutrient intake (NI)): IUGR/ANI, NBW/ANI, IUGR/HNI, and NBW/HNI (*n* = 6, per group). The ANI formula milk was made by mixing 1 kg of formula powder with 4 l of water, whereas HNI formula milk was made by mixing 1.73 kg of formula powder with 4 l of water, whose nutrient contents were about 1.5-fold of the ANI. The basic formula milk powder was formulated according to our previous study [12], and the composition was shown in Additional file 1: Table S1. One hundred milliliters of ANI formula milk contained 5.06 g protein, 4.64 g lactose, and 5.20 g lipids, which were similar to that in the same volume of sow milk, containing 5.00 g protein, 5.06 g lactose, and 7.90 g lipids [12]. One hundred milliliters of HNI formula milk contained 7.59 g protein, 6.96 g lactose, and 7.80 g lipids. All pigs were fed with corresponding formula milk at 50 ml/kg body weight (BW) per meal with a feeding bottle seven times per day at 3-h intervals between 06:00 and 24:00. Pigs had free access to water. The ambient temperature and humidity were controlled around 30 °C and 50~60%, respectively. This experiment lasted for 21 days.

### Tissue sample collection

At the end of the trial, all pigs were anesthetized with an intravenous injection of pentobarbital sodium (15 mg/kg BW) and killed. A set of morphometric measurements were made: head length (snout to between ears, HL), crow-rump length (between ears to end of tail; CRL), and abdominal circumference (AC), before slaughtering. Body mass index (BMI; BW/CRL^2^) was calculated for each pig. The brain and heart of each pig were weighed immediately. The semitendinosus (ST) muscle and psoas major (PM) muscle from the left side of each carcass were completely excised and weighed, and the length and the circumference of the mid belly muscles were recorded. Muscle samples for histological analyses were excised from the central region of PM muscle, and then stored in 4% methanol solution. In addition, other muscle samples of PM muscle were collected, snap-frozen in liquid nitrogen, and stored at − 80 °C.

### Muscular morphology

The PM muscle samples stored in 4% methanol solution were prepared after staining with hematoxylin and eosin using standard paraffin-embedding procedures. All sections were photographed using a digital microscope (Nikon), and muscle fibers were counted over five randomly selected fields of known size (1.01 mm^2^, 200–300 fibers) as the myofiber density [17]. Muscle CSA was calculated from the circumference of the mid belly muscle. Then, the estimated total myofiber number was obtained by multiplying the fiber number per unit area by the CSA of PM muscle. The myofiber density was used to estimate the total number of fiber by multiplying with the CSA of PM muscle. The mean muscle fiber diameter in the united area was measured by Image-Pro Plus 6.0 software (Media Cybernetics, Bethesda, MD).

### Biochemical analyses

Total RNA of PM muscle samples was extracted using TRIzol reagent (Invitrogen, USA) according to the manufacturer’s instruction. RNA concentration and quality were verified by both spectrometry and agarose gel (1.0%) electrophoresis. DNA of muscle samples was extracted using the QIAamp® DNA mini kit (Qiagen) according to the manufacturer’s instructions, and DNA quantification was performed using a NanoVue Plus spectrophotometer (GE Lifescience, Piscataway, NJ, USA). Protein concentration, lactate dehydrogenase (LDH), citrate synthase (CS), and β-hydroxy-acyl-CoA-dehydrogenase (HAD) activities of muscular samples were determined by using a commercial kit (Nanjing Jiancheng Bioengineering Institute, Nanjing, China) according to the instruction manuals. Briefly, frozen muscle samples (approximately 50 mg) were homogenized in 450 μl of 0.9% saline and then centrifuged at 3500*g* for 10 min at 4 °C. The protein content in muscle supernatant was determined based on the method of Coomassie brilliant blue dyeing using bovine serum albumin as the standard. The rate of change of absorbance was monitored at 440, 340, and 412 nm for evaluation of LDH, CS, and HAD activity using a biochemical analyzer (Multiskan Spectrum, Thermo Scientific), respectively. Their activities were expressed as units per gram of protein (U/g protein).

### Real-time reverse transcription-PCR (RT-PCR)

Reverse transcription was performed at 37 °C for 15 min, followed by RT inactivation at 85 °C for 5 s using PrimeScript™ RT reagent Kit (Catalog no. RR047A; Takara). A portion of the RT products (1 μl) was used directly for real-time PCR. Real-time PCR assays were performed on complementary DNA samples in 384 well-optical plates on a 7900HT ABI Prism Sequence Detection System (Applied Biosystems, Foster City, CA, USA) using the SYBR green system (Catalog no. RR820A; Takara). Primers for individual genes were designed using Primer Express 3.0 (Applied Biosystems) and given in Table 1. The reaction mixture (10 μl) contained 5 μl of fresh SYBR® *Premix Ex* TaqII (Tli RNaseH Plus) and 0.2 μl ROX Reference Dye II (50×), 0.8 μl of the primers, 1 μl of RT products, and 3 μl dH_2_O. The PCR protocol was used as follows: 1 cycle (95 °C 30 s), 40 cycles (95 °C 5 s, 60 °C 31 s), and 1 cycle (95 °C 15 s, 60 °C 1 min and 95 °C 15 s). The standard curve of each gene was run in duplicate and three times for obtaining reliable amplification efficiency values as described previously [18]. The correlation coefficients (*r*) of all the standard curves were more than 0.99, and the amplification efficiency values were between 90 and 110%. At the end of amplification, dissociation analyses of the PCR product were performed to confirm the specificity of PCR products. The relative mRNA abundance of analyzed genes was calculated using the method of 2^−ΔΔCt^ [19], and 18S rRNA was used as a reference gene in this study. In addition, the percentages of MyHC isoforms was calculated as the ratio of the normalized expression level of each MyHC isoform to the total expression of MyHC [20].

**Table 1:** Primer sequences of target and reference genes

Gene	Primer sequence (5′–3′)	Product size (bp)	Genbank ID
*IGF-1*	F: GAACTGAAGAGCGTCCACCA	81	NM_214256.1
	R: TGCTTGCTCTCCTTCACCAG		
*IGF-1R*	F: ATGGATCACAAAGCCCTCGG	148	HQ322390.1
	R: CTGCCGCCACTACTACTACG		
*GHR*	F: GCTGTATGGATCCAGGGCTC	144	NM_214254.2
	R: TGCAGAGAGTTCATCCAGGC		
*Akt*	F: TCCAGCTTGAGGTCCCGATA	132	NM_001159776.1
	R: GCTCTTCTTCCACCTGTCCC		
*mTOR*	F: GGGGTTTGGATCAGGGTCTG	80	XM_003127584.4
	R: GACTCATCCGCCCCTACATG		
*RPS6K*	F: TTGAACTTCTCCAGCGTCCC	106	XM_003131671.3
	R: GCCTCCCTACCTCACACAAG		
*RPS6*	F: TACTCAGTAGCAGGCGGACT	92	XM_005660083.1
	R: TACTCAGTAGCAGGCGGACT		
*eIF4E*	F: ATGGAAGTCACTGTGGCCTG	133	DQ826509.1
	R: TCGTCCCACTAGCTCACAGA		
*eIF4EBP1*	F: CACAGGTGAGTTCCGACACT	105	NM_001244225.1
	R: GACTACAGCACCACTCCCG		
*MyHC I*	F: AAGGGCTTGAACGAGGAGTAGA	130	AB053226
	R: TTATTCTGCTTCCTCCAAAGGG		
*MyHC IIa*	F: GCTGAGCGAGCTGAAATCC	155	AB025260
	R: ACTGAGACACCAGAGCTTCT		
*MyHC IIb*	F: ATGAAGAGGAACCACATTA	137	AB025261
	R: TTATTGCCTCAGTAGCTTG		
*MyHC IIx*	F: AGAAGATCAACTGAGTGAACT	113	AB025262
	R: AGAGCTGAGAAACTAACGTG		
*18S rRNA*	F: GACTCAACACGGGAAACCTCAC	146	AY265350.1
	R: ATCGCTCCACCAACTAAGAACG		

*IGF-1* insulin-like growth factor 1, *IGF-1R* insulin-like growth factor 1 receptor, *GHR* growth hormone receptor, *Akt* protein kinase B, *mTOR* mammalian target of rapamycin, *RPS6K* ribosomal protein S6 kinase, *RPS6* ribosomal protein S6, *eIF4E* eukaryotic translation initiation factor 4E, *eIF4EBP1* eukaryotic translation initiation factor 4E binding protein 1, *MyHC* myosin heavy chain

### Western blotting

Western blot analysis was performed as previously described [21]. For the preparation of protein lysates, frozen muscle samples were powdered under liquid nitrogen and was homogenized in 1 ml cell lysis buffer (Beyotime Biotechnology, Shanghai, China) supplemented with protease inhibitor cocktail (Roche, Mannheim, Germany) on a homogenizer. The protein lysate was centrifuged at 12,000*g* and 4 °C for 30 min, and the supernatant was transferred to a new EP tube. The concentration of protein in the supernatant was measured with a BCA Protein Assay Kit (Thermo). One hundred micrograms protein was used to prepare an electrophoresis sample with loading buffer (Bio-Rad, Shanghai, China) in a volume of 30 μl for each sample. Proteins were separated on 12% polyacrylamide gel, and then transferred onto polyvinylidene fluoride membranes (Bio-Rad Laboratories). The membranes were blocked in 1% BSA/1 × TBST for 1 h at room temperature, followed by incubation with the appropriate primary antibodies overnight. Total mTOR (1:1000; Santa Cruz), phosphorylated mTOR (Ser^2448^, 1:1000 dilution), total eIF4E (1:1000 dilution), and phosphorylated eIF4E (Ser^209^, 1:1000 dilution) were obtained from Santa Cruz (Shanghai, China); total RPS6 (1:2000 dilution), phosphorylated RPS6 (Ser^235/236^, 1:1000 dilution), total Akt (1:1000 dilution), phosphorylated Akt (Ser^473^, 1:1000 dilution), and β-actin (1:500 dilution) were obtained from Cell Signaling Technology (Shanghai, China). The membrane was then washed with 1× TBST for three times at 4 °C. After thorough washing, membranes were incubated with appropriate horseradish peroxidase-linked secondary antibodies (Cell Signaling Technology, Shanghai, China) (1:2000 dilution in 5% milk/1× TBST) for 1 h. After further thorough washing, protein signals were detected by ECL western blotting detection reagent (Bio-Rad, Shanghai, China) on a Molecular Imager ChemiDoc XRS+ System (Bio-Rad Laboratories). Blots were quantified with ImageJ software (National Institutes of Health, Bethesda, MD).

### Statistical analysis

Results are presented as means with their standard errors (SEM). Analysis of variance using the General Linear Model (GLM) procedure of SPSS statistical software (Ver.20.0 for Windows, SPSS, Chicago, IL, USA) in the following model: *y*_*ijk*_ = *μ* + *a*_*i*_ + *b*_*j*_ + (*ab*)_*ij*_ + *e*_*ijk*_ (_*i*_ = 1, 2, _*j*_ = 1, 2, _*k*_ = 1, 2,…, *n*_*ij*_), where *y*_*ijk*_ represents the dependent variable, *μ* is the mean, *a*_*i*_ is the effect of BW (IUGR, NBW), *b*_*j*_ is the effect of NI (ANI, HNI), (*ab*)_*ij*_ is the interaction between BW and NI, and *e*_*ijk*_ is the error term. The normality and homogeneity of variances were evaluated by Shapiro–Wilk W test and Levene’s test respectively. Differences were considered as significant when *P* < 0.05, and a tendency was recognized when 0.05 < *P* < 0.10. When significant main effects or interactive effects were observed, the means were compared using Tukey’s multiple comparisons with a *P* < 0.05 indicating significance.

## Results

### Organ indices

IUGR markedly decreased heart weight, AC, and CRL (*P* < 0.05), but increased the ratio of brain to BW and the ratio of HL to BW (*P* < 0.05) (Table 2). HNI increased heart weight, AC, and BMI (*P* < 0.05), but decreased the ratio of brain to BW and the ratio of HL to BW (*P* < 0.05) regardless of BW. In addition, no interaction between BW and NI was found on organ indices. Furthermore, compared with NBW pigs receiving ANI, the heart weight, AC, and BMI were higher (*P* < 0.05), but the relative brain weight and the ratio of HL to BW were lower (*P* < 0.05) in NBW pigs receiving HNI, respectively.

**Table 2:** Effects of the level of nutrient intake on the organ weights, organ to body weight ratios, and morphometry of intrauterine growth-restricted (IUGR) and normal birth weight (NBW) pigs

Items	ANI		HNI		SEM	*P* value		
	IUGR	NBW	IUGR	NBW		BW	NI	BW × NI
Brain, g	58.0	60.4	63.3	56.7	7.2	0.585	0.837	0.255
Heart, g	31.1^a^	42.3^b^	36.4^a^	50.6^c^	3.8	< 0.001	0.003	0.439
Brain: BW, %	1.05^c^	0.82^b^	0.94^b,c^	0.64^a^	0.11	0.001	0.025	0.573
Heart: BW, %	0.56	0.57	0.54	0.57	0.05	0.406	0.605	0.559
HL, cm	17.8	18.5	18.8	17.0	2.1	0.632	0.801	0.272
AC, cm	35.4^a^	42.1^b^	37.8^a^	45.5^c^	1.9	< 0.001	0.012	0.608
CRL, cm	43.6^a^	48.3^a,b^	46.0^a,b^	51.3^b^	3.4	0.017	0.871	0.161
BMI, kg/m^2^	29.1^a^	28.2^a^	33.3^a,b^	38.4^b^	4.59	0.431	0.016	0.268
HL:BW, cm/kg	3.2^c^	2.5^b^	2.8^b,c^	1.9^a^	0.36	0.001	0.011	0.723

Data are presented as mean values with their standard errors, *n* = 6 in each group. Mean values within a row with different superscript letters (a, b, c) were significantly different between four groups (*P* < 0.05)

*ANI* adequate nutrient intake, *HNI* high nutrient intake, *BW* body weight, *NI* nutrient intake, *HL* head length, *AC* abdominal circumference, *CRL* crown-rump length, *BMI* body mass index

### Muscle weight

IUGR significantly decreased the weight, length, and the relative weight to BW of both ST and PM muscles (*P* < 0.05) (Table 3), while increased the ratio of brain to ST weight and the ratio of brain to PM weight of pigs (*P* < 0.05). HNI increased the weight and length of PM muscle (*P* < 0.05) but decreased the ratio of brain to PM muscle weight (*P* < 0.05). Although not statistically significant, HNI tended to exhibit longer ST (*P* = 0.095) and greater (*P* = 0.085) ratio of PM weight to BW. Compared with IUGR pigs receiving ANI, the PM weight and length were higher (*P* < 0.05), but the ratio of brain to PM was lower (*P* < 0.05) in IUGR pigs receiving HNI, respectively.

**Table 3:** Effects of the level of nutrient intake on the muscle weights and lengths, muscle to body weight ratios, and brain to muscle ratios of intrauterine growth-restricted (IUGR) and normal birth weight (NBW) pigs

Items	ANI		HNI		SEM	*P* value		
	IUGR	NBW	IUGR	NBW		BW	NI	BW × NI
ST weight, g	16.5^a^	27.3^b^	19.9^a^	30.8^b^	4.52	< 0.001	0.167	0.972
ST length, cm	5.8^a^	6.9^b^	6.7^a,b^	7.3^b^	0.68	0.028	0.095	0.471
ST: BW, %	0.30	0.37	0.29	0.35	0.06	0.048	0.662	0.752
Brain: ST	3.66^b^	2.21^a^	3.31^b^	1.92^a^	0.60	0.001	0.320	0.921
PM weight, g	15.3^a^	23.9^b^	21.5^b^	30.0^c^	2.46	< 0.001	< 0.001	0.995
PM length, cm	7.5^a^	9.5^b^	9.4^b^	10.6^b^	0.93	0.005	0.006	0.416
PM: BW, %	0.28^a^	0.32^a,b^	0.32^a,b^	0.34^b^	0.03	0.048	0.085	0.429
Brain: PM	3.81^c^	2.57^b^	2.97^b^	1.90^a^	0.38	< 0.001	0.002	0.666

Data are presented as mean values with their standard errors, *n* = 6 in each group. Mean values within a row with different superscript letters (a, b, c) were significantly different between four groups (*P* < 0.05)

*ANI* adequate nutrient intake, *HNI* high nutrient intake, *BW* body weight, *NI* nutrient intake, *ST* semitendinosus, *PM* psoas major

### Muscle characteristics

IUGR decreased the CSA and myofiber number (*P* < 0.05) in the PM muscle of pigs, respectively (Table 4). Although not statistically significant, moreover, IUGR pigs tended to show higher myofiber density of PM (*P* = 0.083) than NBW pigs. HNI increased the CSA (*P* < 0.05) and tended to decrease myofiber density of PM muscle (*P* = 0.054) regardless of BW. BW and NI had a significant interaction effect on the CSA and myofiber density of PM muscle (*P* < 0.05). The CSA of PM was higher (*P* < 0.05) while the myofiber density was lower (*P* < 0.05) in NBW pigs receiving HNI, respectively, compared with NBW pigs receiving ANI.

**Table 4:** Effects of the level of nutrient intake on histomorphometry of psoas major muscle of intrauterine growth-restricted (IUGR) and normal birth weight (NBW) pigs

Items	ANI		HNI		SEM	*P* value		
	IUGR	NBW	IUGR	NBW		BW	NI	BW × NI
CSA, mm^2^	156.2^a^	209.1^b^	176.2^a,b^	344.8^c^	30.7	< 0.001	< 0.001	0.003
Myofiber diameter, um	24.7	23.4	23.3	23.6	2.4	0.720	0.663	0.555
Myofiber density, mm^2^	2161^b^	2270^b^	2227^b^	1463^a^	338	0.083	0.054	0.027
Myofiber number, thousand	337.2^a^	461.6^b^	394.6^a,b^	498.7^b^	68.1	0.004	0.178	0.764

Data are presented as mean values with their standard errors, *n* = 6 in each group. Mean values within a row with different superscript letters (a, b, c) were significantly different between four groups (*P* < 0.05)

*ANI* adequate nutrient intake, *HNI* high nutrient intake, *BW* body weight, *NI* nutrient intake, *CSA* cross-sectional area

### Biochemical properties

IUGR had no influence on RNA, DNA, and protein concentrations of PM muscle (Table 5). HNI markedly increased the concentrations of RNA and the ratio of RNA to DNA in PM muscles (*P* < 0.05) regardless of BW. Although not statistically significant, HNI tended to increase the concentration of protein in the PM muscle (*P* = 0.082). Compared with NBW pigs receiving ANI, the RNA concentration of PM was higher (*P* < 0.05) in IUGR pigs receiving HNI.

**Table 5:** Effects of the level of nutrient intake on the concentrations of RNA, DNA, and protein in psoas major muscle of intrauterine growth-restricted (IUGR) and normal birth weight (NBW) pigs

Items	ANI		HNI		SEM	*P* value		
	IUGR	NBW	IUGR	NBW		BW	NI	BW × NI
RNA, mg/g	1.68^a,b^	1.63^a^	2.05^b^	1.79^a,b^	0.23	0.209	0.039	0.392
DNA, mg/g	1.50	1.40	1.48	1.38	0.20	0.292	0.884	0.987
Protein, mg/g	181.6	173.9	186.0	190.6	12.6	0.725	0.082	0.248
RNA: DNA	1.14	1.18	1.39	1.30	0.19	0.828	0.047	0.478
Protein: DNA	123.3	126.5	139.7	126.4	15.7	0.353	0.350	0.611

Data are presented as mean values with their standard errors, *n* = 6 in each group. Mean values within a row with different superscript letters (a, b) were significantly different between four groups (*P* < 0.05)

*ANI* adequate nutrient intake, *HNI* high nutrient intake, *BW* body weight, *NI* nutrient intake

### Metabolic enzyme activities

**Fig. 1:**
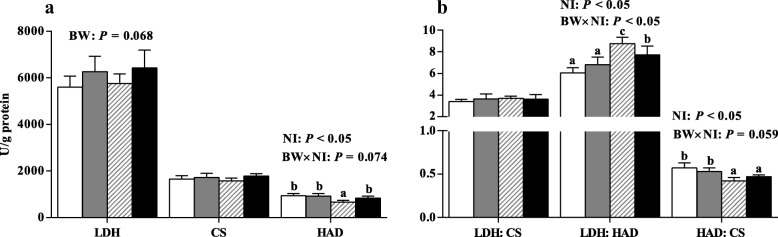
Effects of the level of nutrient intake on activities of LDH, CS, and HAD (**a**) and the ratio of LDH:CS, LDH:HAD, and HAD:CS (**b**) in psoas major muscle of pigs with different birth weights. Values are means, with their standard errors represented by vertical bars (*n* = 6 for each group). Mean values with different letters (a, b, c) were significantly different between four groups (*P* < 0.05). , IUGR/ANI; , NBW/ANI; , IUGR/HNI; , NBW/HNI. *ANI*, adequate nutrient intake; *HNI*, high nutrient intake; *IUGR*, intrauterine growth restriction; *NBW*, normal birth weight; *LDH*, lactate dehydrogenase; *CS*, citrate synthase; *HAD*, β-hydroxy-acyl-CoA-dehydrogenase

IUGR tended to decrease the LDH activity of PM (Fig. 1, *P* = 0.068) although not statistically significantly. Regardless of BW, HNI increased the ratio of LDH to HAD (*P* < 0.05) but decreased the activity of HAD and the ratio of HAD to CS in the PM muscle (*P* < 0.05). BW and NI had significant interaction effect on the ratio LDH to HAD (*P* < 0.05) in the PM muscle. Compared with IUGR pigs receiving ANI, the HAD activity and the ratio of HAD to CS were lower (*P* < 0.05), but the ratio of LDH to HAD was higher (*P* < 0.05) in IUGR pigs receiving HNI, respectively.

### Messenger RNA expression

**Fig. 2:**
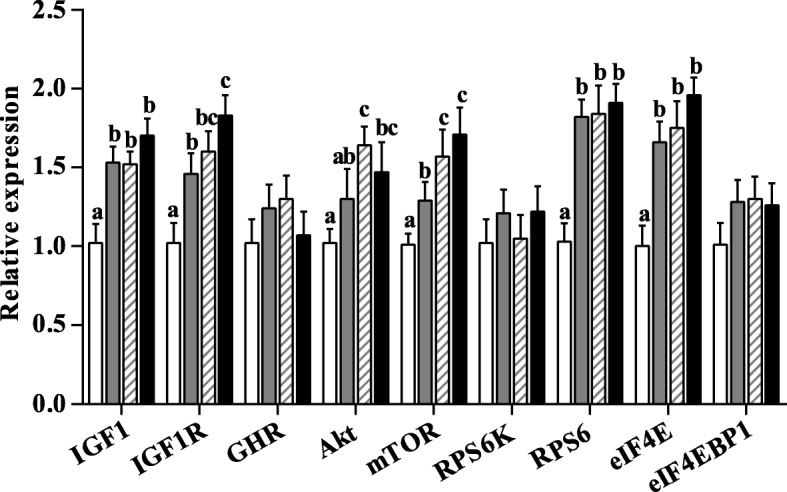
Effects of the level of nutrient intake on mRNA abundance of growth-related genes in psoas major muscle of pigs with different birth weight. Values are means, with their standard errors represented by vertical bars (*n* = 6 for each group). Mean values with different letters (a, b, c) were significantly different between four groups (*P* < 0.05). , IUGR/ANI; , NBW/ANI; , IUGR/HNI; , NBW/HNI. *ANI*, adequate nutrient intake; *HNI*, high nutrient intake; *IUGR*, intrauterine growth restriction; *NBW*, normal birth weight; *IGF1R*, insulin-like growth factor 1 receptor; *GHR*, growth hormone receptor; *Akt*, protein kinase B; *mTOR*, mammalian target of rapamycin; *RPS6*, ribosomal protein S6; *eIF4E*, eukaryotic translation initiation factor 4E; *eIF4EBP1*, eukaryotic translation initiation factor 4E binding protein 1. For IGF1, IGF1R, mTOR, RPS6, and eIF4E, there was a significant effect of body weight (*P* < 0.05). For IGF1, IGF1R, Akt, mTOR, RPS6, and eIF4E, there was a significant effect of nutrient intake (*P* < 0.05). Furthermore, there was a significant interaction between body weight and nutrient intake on the mRNA abundance of GHR and RPS6 (*P* < 0.05)

To clarify the mechanism involved in protein synthesis in the PM muscle of pigs, we measured the expression of gene-related protein synthesis. IUGR pigs had significantly decreased the mRNA abundance of IGF1, IGF1R, mTOR, RPS6, and eIF4E (*P* < 0.05) in the PM muscle relative to NBW pigs (Fig. 2). Regardless of BW, HNI significantly increased the mRNA abundance of IGF1, IGF1R, Akt, mTOR, RPS6, and eIF4E in the PM muscle (*P* < 0.05). Significant interaction effects were found between BW and NI on the mRNA abundance of GHR and RPS6 (*P* < 0.05) in the PM muscle. BW and NI had a significant interaction effect on the mRNA abundance of RPS6 (*P* < 0.05). Compared with IUGR pigs receiving ANI, the mRNA abundance of IGF1, IGF1R, Akt, mTOR, RPS6, and eIF4E were higher in the PM of IUGR pigs receiving HNI (*P* < 0.05).

### The composition of myofiber type

**Fig. 3:**
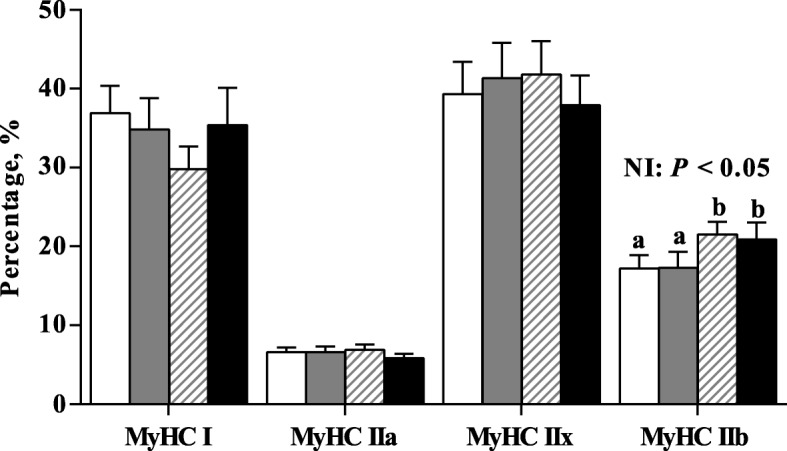
Effects of the level of nutrient intake on the percentage of MyHC in psoas major muscle of pigs with different birth weights. Values are means, with their standard errors represented by vertical bars (*n* = 6 for each group). Mean values with different letters (a, b) were significantly different between four groups (*P* < 0.05). , IUGR/ANI; , NBW/ANI; , IUGR/HNI; , NBW/HNI. *ANI*, adequate nutrient intake. *HNI*, high nutrient intake; *IUGR*, intrauterine growth restriction; *NBW*, normal birth weight; *MyHC*, myosin heavy chain

HNI increased the percentage of MyHC IIb in the PM muscle (*P* < 0.05) of pigs (Fig. 3). No significant changes in the percentages of other MyHC were found between groups (*P* > 0.05). The percentage of MyHC IIb in the PM muscle was higher (*P* < 0.05) in IUGR pigs receiving HNI compared with IUGR pigs receiving ANI.

### Protein expressions

**Fig. 4:**
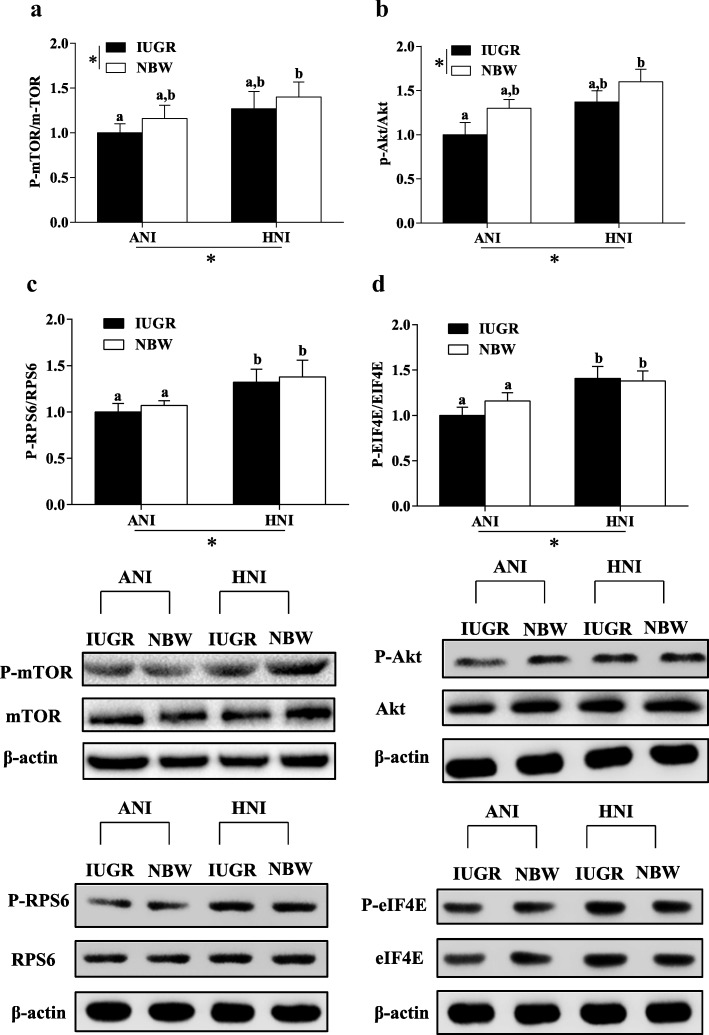
Effects of the level of nutrient intake on abundance of growth-related proteins in psoas major muscle of pigs with different birth weight. *ANI*, adequate nutrient intake; *HNI*, high nutrient intake; *IUGR*, intrauterine growth restriction; *NBW*, normal birth weight; *mTOR*, mammalian target of rapamycin; *p-mTOR*, phosphorylated mTOR; *Akt*, protein kinase B; *P-Akt*, phosphorylated Akt; *RPS6*, ribosomal protein S6; *P-RPS6*, phosphorylated RPS6; *eIF4E*, eukaryotic translation initiation factor 4E; *P-eIF4E*, phosphorylated eIF4E. Values are means, with their standard errors represented by vertical bars (*n* = 6 for each group). Mean values with different letters (a, b) were significantly different between four groups (*P* < 0.05). ^*^P < 0.05 for the respective sources of variation (nutrition intake or body weight)

We measured the protein expression of IGF-1-Akt-mTOR signal pathway in the PM muscle. IUGR pigs had significantly decreased the protein expression of P-mTOR (Fig. 4a) and P-Akt (Fig. 4b) in the PM muscle relative to NBW pigs (*P* < 0.05). Regardless of BW, HNI significantly increased the protein expressions of P-mTOR (Fig. 4a), P-Akt (Fig. 4b), P-RPS6 (Fig. 4c), and P-eIF4E (Fig. 4d) in the PM muscle of pigs (*P* < 0.05). Compared with IUGR pigs receiving ANI, the protein expression of P-RPS6 (Fig. 4c) and P-eIF4E (Fig. 4d) were higher (*P* < 0.05) in IUGR pigs receiving HNI.

## Discussion

Previous studies have been shown that neonates with IUGR receiving high-density nutrition intake have high risk of adult onset metabolic diseases, such as type 2 diabetes and cardiovascular disease [22, 23]. Skeletal muscle plays important roles in many physiological activities and is vital for contributing to basal metabolic rate of body [24]. However, little is known about the effects of HNI during the early postnatal period on skeletal muscle growth and metabolic status or its potential mechanism in IUGR neonates. The major finding of this study is that HNI during the early postnatal period improved the skeletal growth through mechanisms stimulating protein synthesis in IUGR pigs. HNI exerted marked differential effects on metabolic status and muscle properties; these changes reflected catch-up development induced by HNI during the early postnatal period. These findings have important implications for long-term muscle development of human infants with IUGR.

Neonates with IUGR had lighter body weight and increased relative internal organ weights [25], which is consistent with the observed shorter AC and CRL of IUGR pigs compared with that of NBW pigs in our study. By contrast, IUGR pigs had a markedly higher relative brain weight than that of NBW pigs, indicating that the maintenance of brain weight is of primary importance for IUGR pigs [26]. This phenotype could be due to the metabolic priority for the growth of key organs when fetus suffering maternal malnutrition in uterus [27]. The increased BMI and heart weight in IUGR pigs with HNI may be resulting from the compensatory growth during the early postnatal period, which is in accordance with the report of Greenwood et al., who showed that IUGR sheep with a high postnatal nutrition resulted in a greater internal organ weight [28].

Numerous studies had demonstrated that pigs suffering IUGR had smaller muscle mass [29, 30], reflecting decreased muscle growth prenatally and/or postnatally [6, 31]. In the present study, IUGR decreased the PM and ST muscle weight of pigs, which are in agreement with previous findings [32]. However, we found that there was a comparable PM muscle weight between IUGR pigs with HNI and NBW pigs with ANI. The growth performance found in our previous study showed IUGR pigs receiving HNI were able to exert a similar growth rate and comparable weight gain to NBW pigs with ANI [12].

Neonates suffering IUGR formed a lower myofiber number which will restrict the potential of lean growth after birth [4]. In our study, both the myofiber number and CSA of PM muscle were lower in IUGR pigs than NBW pigs, which are in agreement with previous reports [4, 5, 29]. Consistent with pervious study [32], IUGR pigs had a higher myofiber density than their normal littermate. However, HNI tended to decrease the myofiber density during the suckling period, which may result from increased CSA. The number and CSA of muscle fiber are known to be an important determinant of postnatal muscle growth [33]. Total myofiber number of pigs has been reported to be fixed at birth [34, 35], but most recently, many studies have found that there might be a “third generation” for muscle fiber growth during the early postnatal period [36–38], which are shown by the occurrence of very small-diameter myofibers containing embryonic and/or neonatal isoforms of myosin heavy chain in porcine muscles [39]. Interestingly, compared with NBW pigs, a comparable myofiber number of PM muscle in IUGR pigs were found when receiving HNI, which suggests that the restricted myofiber of pigs suffering IUGR could be induced by HNI during the early postnatal period. Similarly, Losel et al. reported that L-carnitine supplementation also increased the myofiber number of IUGR pigs during the suckling period [40]. Ratios of protein to DNA and RNA to DNA have been used as indexes of hypertrophy and potential cellular activity respectively [41]. In the current study, the early postnatal fiber formation of IUGR pigs in response to HNI was associated with greater RNA concentrations and the ratio of RNA to DNA, as well as tended to increase the concentration of protein in the PM muscle, which could be explained as a shift to intensified myogenic proliferation [40]. These results provide another line of evidence for the increased myofiber number of IUGR pigs receiving HNI during the suckling period. Taken together, these results indicated the myofiber formation in IUGR pigs has not fully ceased at birth and can be improved by HNI in the early postnatal phase.

Muscle development in postnatal pigs is regulated by gene expression and growth factors [42]. IGF-1 acts as an important mediator to promote cell differentiation and protein synthesis in fetuses [43]. Skeletal muscle growth in response to IGF-1 is precisely mediated by the serine/threonine kinase Akt, the downstream targets of mTOR signal pathway, through regulation of mRNA translation and protein synthesis [11, 44]. Consistent with previous studies [45, 46], the decreased mRNA abundance of IGF1, IGF1R, mTOR, RPS6, and eIF4E and protein expression of P-mTOR and P-Akt suggested that the muscle growth is compromised in pigs suffering IUGR. However, our results demonstrated that HNI improved muscle growth via IGF1-Akt-mTOR signal pathway, as indicated by the enhanced expressions at the transcriptional level. Moreover, Akt activation is reported to stimulate protein synthesis [47]. Therefore, the increased Akt phosphorylation by HNI may be responsible for enhancing the downstream effector. mTOR, as an essential component of the signaling pathway that regulates protein synthesis, is directly activated by the intake of nutrients [48]. Additionally, phosphorylation of mTOR on Ser2448 is positively related to the activity of mTOR [49]. Consistent with this observation, we found that HNI resulted in an increase of the protein expressions of P-mTOR in the PM muscle of pigs, suggesting that HNI during the early postnatal period enhanced the activation of elements involved in the mTOR protein synthesis pathway. Moreover, the increased P-RPS6 and P-eIF4E by HNI (the downstream effector of mTOR) supported this conclusion. eIF4E phosphorylation status was examined because it influences mRNA bindings to the 43S preinitiation complex, resulting in increased protein synthesis in cell culture [50]. Zhu et al. has reported that restricted nutrient intake affected the protein expressions by mTOR-RPS6 pathway in the muscle of sheep [51].

Rapid myofibril protein accretion and changes in MyHC polymorphism of myofibers in the skeletal muscle of pigs have been reported during the neonatal period [52]. In the present study, we found that HNI increased the percentage of MyHC IIb-type muscle fiber in the PM muscle of both IUGR and NBW pigs. Studies have suggested that high muscularity is positively related to a high abundance of MyHC IIb transcript [20]; the MyHC IIb mRNA levels had been shown to be steadily increased from days 7 to 180 after birth. Furthermore, the phenotypic changes in muscle characteristics are often linked to the metabolic and functional alterations [11]; therefore, the activities of related metabolic enzymes were measured in the present study. The activities of CS, HAD, and LDH were used as markers of overall oxidative capacity (tricarboxylic cycle), lipid β-oxidation, and glycolytic potential, respectively [7]. In the present study, we found that IUGR tended to decrease the activity of LDH in the PM muscle of pigs, which suggested that glycolytic muscles might be more susceptible to IUGR. However, the ratio of LDH to HAD in the PM muscle of pigs was enhanced by HNI, which is indicative of increased glycolytic capacity of skeletal muscle when pigs are receiving HNI [14]. Moreover, a shift toward a lower lipid β-oxidation capacity was observed in the PM muscle of pigs receiving HNI, as suggested by the decreased HAD activity and the ratio of HAD to CS. Most importantly, IUGR pigs receiving HNI had a higher ratio of LDH to HAD than those receiving ANI, suggesting that restricted glycolytic capacity could be restored by HNI during the early postnatal period. Similarly, Lefaucheur et al. has shown that high food intake during the early postnatal period enhanced the muscular glycolytic capacity, as indicated by the increased ratio of LDH to HAD in the muscle of pigs [14], the increased glycolytic enzyme activity could be observed in the early postnatal phase during the muscular development [53]. Based on the data of metabolic enzymes and MyHC isoform, HNI during the suckling period may accelerate the muscle maturity of pigs.

## Conclusion

In summary, the results of the current study indicate that high nutrient intake during the early postnatal period contributed to skeletal muscle fiber growth and maturity through stimulating protein synthesis-related gene expression and accelerating glycolytic fiber development in IUGR pigs, associated with changing the metabolic status, which indicated the long-term effect of IUGR on muscle development.

## Additional file

Additional file 1: **Table S1.** Composition and nutrient level of the basal formula milk powder (87.5% DM basis, %). (DOCX 33 kb)
