# Supplementary material for: High nutrient intake during the early postnatal period accelerates skeletal muscle fiber growth and maturity in intrauterine growth-restricted pigs
Source: Genes Nutr. 2018 Jul 27;13:23. doi: 10.1186/s12263-018-0612-8 (PMC6062929; doi:10.1186/s12263-018-0612-8)
Supplement: Supplementary file 1 — Table S1. Composition and nutrient level of the basal formula milk powder (87.5% DM basis, %). (DOCX 33 kb) [file 12263_2018_612_MOESM1_ESM.docx]

**Table S1.** Composition and nutrient level of the basal formula milk powder (87.5 % DM basis, %)

| Ingredients | % |
| --- | --- |
| Whole-milk powder (24% CP) | 58.00 |
| Whey protein concentrate (34% CP) | 25.00 |
| Casein | 5.70 |
| Coconut oil | 10.00 |
| CaH_2_PO_4_ | 0.10 |
| Choline chloride (50%) | 0.10 |
| Vitamin premix^*^ | 0.10 |
| Mineral premix^†^ | 0.50 |
| L-Arg (98.5%) | 0.06 |
| DL-Met (98.5%) | 0.06 |
| L-Lys·HCl (78.5%) |  |
| L-Thr (98%) | 0.03 |
| L-Trp (98%) | 0.05 |
| Total | 100.00 |
| Nutrient content |  |
| Digestible energy (kJ/kg) | 18390 |
| CP (%) | 25.30 |
| Ca (%) | 1.02 |
| Total P (%) | 0.81 |
| Available P (%) | 0.67 |
| Digestible Lys (%) | 1.93 |
| Digestible Met (%) | 0.63 |
| Digestible Arg (%) | 0.86 |

CP, crude protein.

^*^Vitamin premix provided per kg powder diet: vitamin A, 0.94 mg; vitamin D_3_, 0.01 mg; vitamin E, 20 mg; vitamin K_3_, 1 mg; vitamin B_12_, 0.04 mg; riboflavin, 5 mg; niacin, 20 mg; pantothenic acid, 15 mg; folic acid, 1.5 mg; thiamin, 1.5 mg; pyridoxine, 2 mg; biotin, 0.1 mg.

^†^Mineral premix provided per kg powder diet: Zn, 90 mg; Mn, 4.0 mg; Fe, 90 mg; Cu, 6.0 mg; I, 0.2 mg; Se, 0.3 mg.
